# A New Method for Determining the Embedding Dimension of Financial Time Series Based on Manhattan Distance and Recurrence Quantification Analysis

**DOI:** 10.3390/e24091298

**Published:** 2022-09-14

**Authors:** Hanhuai Zhu, Jingjing Huang

**Affiliations:** School of Science, Beijing Information Science and Technology University, Beijing 100101, China

**Keywords:** embedding dimension, Manhattan distance, recurrence plot, recurrence quantification analysis

## Abstract

Identification of embedding dimension is helpful to the reconstruction of phase space. However, it is difficult to calculate the proper embedding dimension for the financial time series of dynamics. By this Letter, we suggest a new method based on Manhattan distance and recurrence quantification analysis for determining the embedding dimension. By the advantages of the above two tools, the new method can calculate the proper embedding dimension with the feature of stability, accuracy and rigor. Besides, it also has a good performance on the chaotic time series which has a high-dimensional attractors.

## 1. Introduction

According to the Takens theory [1], a one-dimensional financial time series must have a suitable time delay [2] and embedding dimension [3,4,5,6]. The phase space reconstruction, which is proposed firstly by Takens, can demonstrate the inherent dynamic characteristics of the time series by selecting the appropriate time delay and embedding dimension. Therefore, phase space reconstruction is an important method for analyzing time series [7,8,9], and it has been widely used in various aspects of society such as chemistry, transportation and geology [10,11,12,13,14].

However, determining the time delay and embedding dimension is a necessary step for phase space reconstruction. At present, the time delay is often determined by the method of average mutual information with high accuracy and good applicability [15], while the embedding dimension was originally solved by the method of false-nearest neighbor (FNN) [16]. However, the method contains subjective parameters and depends strongly on the numbers of data points. Therefore, later, Cao made improvements in the principle of the algorithm [17]. Although Cao’s method makes up for the shortcomings of FNN, it is still not good enough in terms of the stability and accuracy of solving the embedding dimension. In addition, it cannot handle financial chaotic time series with high-dimensional attractors well.

At the same time, the recurrence plot [18,19,20] and its recurrence quantification analysis (RQA) [21,22,23], which are related to the embedding dimension, have been successfully applied in analyzing the dynamic characteristics of complex systems, and have been applied to other fields such as economics and sociology [24,25,26,27,28]. What is more, the measures of RQA have been proven to be correlated with the embedding dimension by Zbilut [29]. However, due to the imperfect calculation method of time delay and other parameters, Zbilut only found a qualitative connection, but did not accurately calculate the quantitative relationship between the RQA and embedding dimension. Thus, after verifying its correctness through numerical experiments, we take it as a measure that can help to judge the embedding dimension of the time series. At the same time, we understand that Manhattan distance is applicable in high-dimensional space. It has a similar calculation effect with the Euclidean distance and maximum norm, and has less calculation time [30]. Thus, we improved the algorithm, which is based on the FNN and Cao by using the Manhattan distance [31,32,33]. Finally, through the improvement of the above two ways, two synergistic measures for determining the embedding dimension are obtained, thereby establishing a new embedding dimension calculation method with strong robustness, accuracy and applicability.

The rest of this letter is organized as follows: Section 2 introduces the methodology, which contains a recurrence plot with its quantification and false-nearest neighbor(FNN) method with Cao’s modification and measures of determining the embedding dimension. Section 3 describes the empirical results. Section 4 offers the conclusions.

## 2. Methodology

### 2.1. Indicator 1

For time series $x_{i}\left(i=1\ldots N\right)$, we can reconstruct it in phase space, and obtain the reconstructed vector $\vec{V_{i}\left(m\right)}=\left(x_{i},x_{i+\tau },\ldots ,x_{i+(m-1)\tau }\right),i=1,2,\ldots ,N-\left(m-1\right)\tau$, where τ represents the time delay, determined by the mutual information method, and *m* represents the embedding dimension. Its calculation method is the problem we will explore next. Originally, Matthew B. Kennel used Equations (1)–(3) as indicators to determine the embedding dimension of the chaotic time series with attractors.

(1) $$\begin{array}{c} R_{m}^{2}\left(i,n\left(i,k\right)\right)=\sum_{k=0}^{m-1}{\left[x\left(i+k\tau \right)-x_{n(i,k)}\left(i+k\tau \right)\right]}^{2},i=1,2,\ldots ,n-\left(m-1\right)\tau . \end{array}$$

(2) $$\begin{array}{c} R_{m+1}^{2}\left(i,n\left(i,k\right)\right)=R_{m}^{2}\left(i,n\left(i,k\right)\right)+{\left[x\left(i+k\tau \right)-x_{n(i,k)}\left(i+k\tau \right)\right]}^{2}. \end{array}$$

(3) $$\begin{array}{c} {\left(\frac{R_{m+1}^{2}\left(i,n\left(i,k\right)\right)-R_{m}^{2}\left(i,n\left(i,k\right)\right)}{R_{m}^{2}\left(i,n\left(i,k\right)\right)}\right)}^{\frac{1}{2}}>R_{tol}. \end{array}$$

Among them, $R_{m}^{2}\left(i,n\left(i,k\right)\right)$ represents the distance between the reconstructed vector and the nearest neighbor when the embedding dimension is *m*, and n(i,k) is an integer, determined by *i* and *k*. By choosing an appropriate $R_{tol}$, the optimal embedding dimension is found when the Equation (3) is greater than $R_{tol}$. However, the disadvantage of this method is that the selection of $R_{tol}$ is subjective, which can easily lead to an inaccuracy of the experiment. Later, Cao introduced a(i,m) and E1(m), and improved it by using the maximum norm. It mainly improves the subjective shortcomings of parameter selection, but at the same time exposes new problems. Because the distance between high-dimensional time series is nonlinear, the maximum norm is not suitable for high-dimensional time series. Thus, we introduced a Manhattan distance, and proved its stability and accuracy in processing time series with high-dimensional embedding dimension through experiments. The Manhattan distance is defined as follows:

For the vector $\vec{c}$ and vector $\vec{d}$. If n=2, the Manhattan distance is the L1-norm, and the distance between the two vectors is $MH\left(\vec{c},\vec{d}\right)=|x_{1}-y_{1}\left|+\right|x_{2}-y_{2}|$; if n>2, the Manhattan distance is $MH\left(\vec{c},\vec{d}\right)=|x_{1}-y_{1}\left|+\right|x_{2}-y_{2}\left|+\ldots +\right|x_{n}-y_{n}|$.

By introducing the Manhattan distance and improving a(i,m), the following formula is obtained:

(4) $$\begin{array}{c} a\left(i,m\right)=\frac{\sum_{k=0}^{m-1}\left|x\left(i+k\tau \right)-x_{n(i,k)}\left(i+k\tau \right)\right|}{\sum_{k=0}^{m}\left|x\left(i+k\tau \right)-x_{n(i,k)}\left(i+k\tau \right)\right|},i=1,2,\ldots ,N-m\tau . \end{array}$$

(5) $$\begin{array}{c} E\left(m\right)=\frac{1}{N-m\tau }\sum_{i=1}^{N-m\tau }a\left(i,m\right). \end{array}$$

where

(6) $$\begin{array}{c} E11(m)=E(m+1)/E(m). \end{array}$$

### 2.2. Indicator 2

In order to conduct the experiment more accurately and ensure the success of the experiment, Kennel proposed Equations (7) and (8) as the test standard, but there are many shortcomings; for example, the experimental results are greatly affected by individual subjectivity and cannot identify the chaotic time series.

(7) $$\begin{array}{c} \frac{R_{m+1}^{\left(i\right)}}{R_{A}}>A_{tol}. \end{array}$$

(8) $$\begin{array}{c} R_{A}^{2}=\frac{1}{N}\sum_{i=1}^{N}{\left[x\left(i\right)-\bar{x}\right]}^{2}. \end{array}$$

where $A_{tol}$ is some threshold, $R_{A}$ is the distance between average value and actual value, and

(9) $$\begin{array}{c} \bar{x}=\frac{1}{N}\sum_{i=1}^{N}x\left(i\right). \end{array}$$

To solve the above shortcomings, Cao introduced a new measure E2(m), which can identify deterministic signals from chaotic signals, as shown in the formula,

(10) $$\begin{array}{c} E2\left(m\right)=\frac{E^{*}\left(m+1\right)}{E^{*}\left(m\right)}. \end{array}$$

(11) $$\begin{array}{c} E^{*}\left(m\right)=\frac{1}{N-m\tau }\sum_{i=1}^{N-m\tau }\left|x_{i+m\tau }-x_{n(i,d)+m\tau }\right|. \end{array}$$

However, the above methods have limitations. Through experiments, we found that when the embedding dimension tends to be stable, the magnitude of change will be very small, and it is impossible to determine when the optimal embedding dimension is obtained. At the same time, Webber Jr found embedding dimension is related to the quantification of the recurrence plot. Recurrence plot is a two-dimensional method that can demonstrate the inherent certainty, correlation, and periodicity of the time series. For the time series x(i),i=1,2,…,N, through selecting the time delay and embedding dimension, it is reconstructed as

(12) $$\left(\begin{array}{c} \begin{array}{c} {\vec{V}}_{1}\\ {\vec{V}}_{2}\\ {\vec{V}}_{3}\\ \;\vdots \\ {\vec{V}}_{n} \end{array} \end{array}\right)=\left(\begin{array}{c} \begin{array}{cccccc} \; & x_{1} & x_{1+\tau }\;\;\; & x_{1+2\tau }\;\;\;\cdots & x_{1+(m-1)\tau } & \\ \; & x_{2} & x_{2+\tau }\;\;\; & x_{2+2\tau }\;\;\;\cdots & x_{2+(m-1)\tau } & \\ \; & x_{3} & x_{3+\tau }\;\;\; & x_{3+2\tau }\;\;\;\cdots & x_{3+(m-1)\tau } & \\ \; & \;\vdots & \;\vdots \;\;\;\;\;\; & \;\vdots \; & \;\vdots \;\;\;\\ \; & x_{N} & x_{N+\tau }\;\;\; & x_{N+2\tau }\;\;\cdots & x_{N+(m-1)\tau } \end{array} \end{array}\right)$$

${\vec{V}}_{i}$ represents the *i*-th state, N=n−(m−1)τ is the total number of recurrence points, m≥2 is the embedding dimension, and τ≥1 is the time-delay.

The recurrence plot is drawn by a distance matrix, and the elements in the distance matrix can be defined by the following formula:

(13) $$\begin{array}{c} R_{ij}=H(\epsilon -||{\vec{V}}_{i}-{\vec{V}}_{j}||),i,j=1,2,\ldots ,N. \end{array}$$

The H(x) function is the Heaviside Function; if x>0, the value of H(x) is 1, if x<0, the value of H(x) is 0, *i* represents the number of rows, and *j* represents the number of columns, when $R_{ij}=1$ is 1, it is represented by black dots in the recurrence plot. Otherwise, it is represented by white dots in the recurrence plot. The threshold ϵ is an empirical value. After the above process, the distance matrix is transformed into a matrix. Finally, the 0−1 matrix is visualized and expressed in the form of a two-dimensional plot.

The determinism (DET) is obtained by quantifying the recurrence plot. It is the ratio between the recurrence points and the total recurrence points of a diagonal structure with a length greater than or equal to 1. When the dynamic behavior of the two systems is weakly correlated or uncorrelated, it will produce a very short diagonal structure, and its basic definition is:

(14) $$\begin{array}{c} DET=\frac{\sum_{l=l_{min}}^{N}lp\left(l\right)}{\sum_{l=1}^{N}lp\left(l\right)}. \end{array}$$

$l_{min}$ represents the length of the diagonal structure in the recurrence plot, $l_{min}=2$, and P(l) represents the probability of the diagonal structure with the length *l* in the recurrence plot.

**Figure 1:**
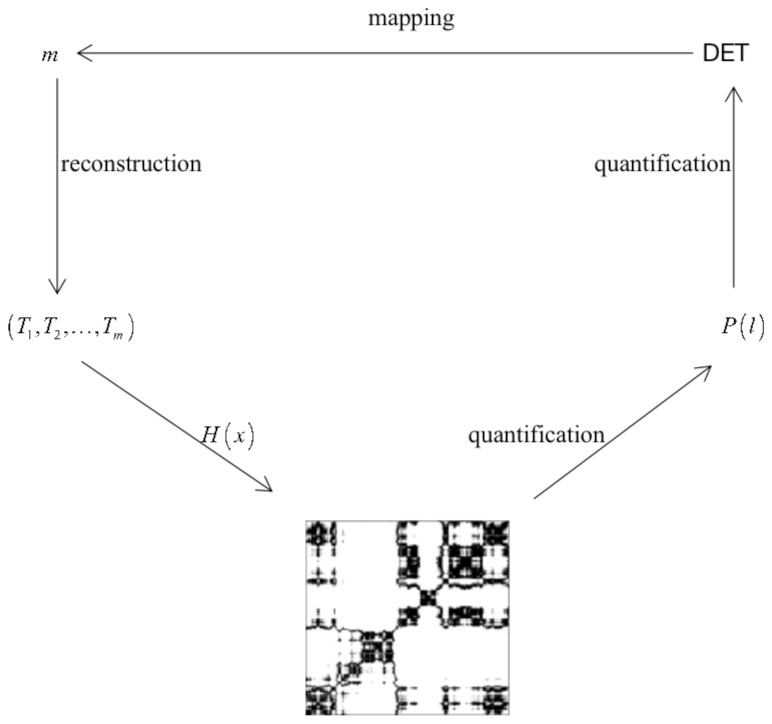
The relationship between the DET and embedding dimension.

After the experiments, we determined the relationship between the DET and embedding dimension. It is demonstrated by the following Figure 1. Based on confirming the correctness of Joseph L.Webber Jr’s view, we proposed a method to assist in determining the embedding dimensions.

## 3. Experiment of Normal Examples

Firstly, we demonstrate the accuracy of our method for solving the embedding dimension. Thus, we selected data with a known minimum embedding dimension of 3.

**Figure 2:**
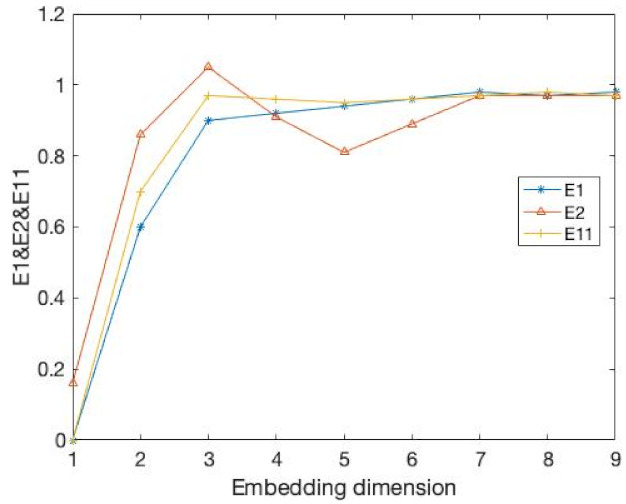
The value of E1, E2 and E11, whose data are from the Lorenz attractor. It reveals uncertain, unrepeatable, and unpredictable chaotic phenomena. E1 and E2 is the measure of Cao’s method. E11 is the measure of our method.

The data are the x-component values from Lorenz attractor with the parameters σ=10, r=28, b=8/3; they are the same as that considered in [9]. We take the integral step equals 0.01, then calculate the numerically integration of the equation. The results are demonstrated in the Figure 2 and Table 1. It is very clear that our method has the accuracy. As the embedding dimension increases, our E11 measure gradually stabilizes. In addition, through the value of DET, we can find that when the embedding dimension is 3, DET, which can help us to determine the embedding dimension, becomes a saturation value when the E11 becomes stable. Therefore, our method can be used to solve the embedding dimension of the time series more accurately.

After discussing the feasibility of our improved method, we next compare the method with the traditional FNN method. As is considered in [9], we select the data from the following equation

(15) $$\begin{array}{c} t_{n+4}=sin\left(t_{n+5}\right)+sin\left(2t_{n+1}+5\right)+sin\left(3t_{n+2}+5\right)+sin\left(4t_{n+3}+5\right). \end{array}$$

By transforming into the following prediction model, we can know that its embedding dimension is 4

(16) $$\begin{array}{c} t_{n+4}=W\left(t_{n},t_{n+1},t_{n+2},t_{n+3}\right)=sin\left(t_{n+5}\right)+sin\left(2t_{n+1}+5\right)+sin\left(3t_{n+2}+5\right)+sin\left(4t_{n+3}+5\right). \end{array}$$

**Figure 3:**
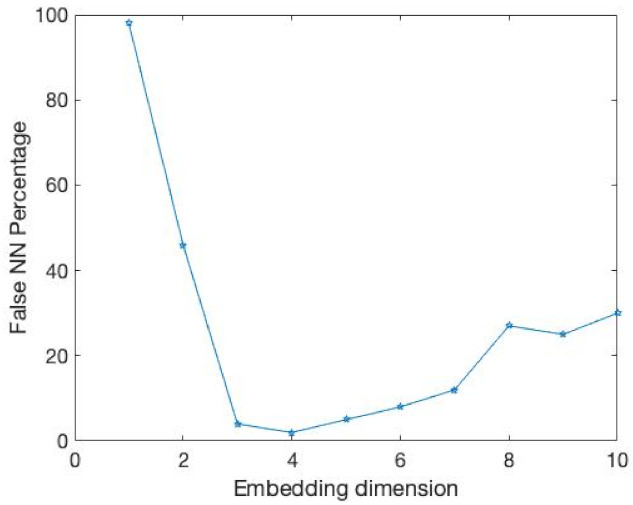
It is the percentage of FNN for the data from above Equation (15).

**Figure 4:**
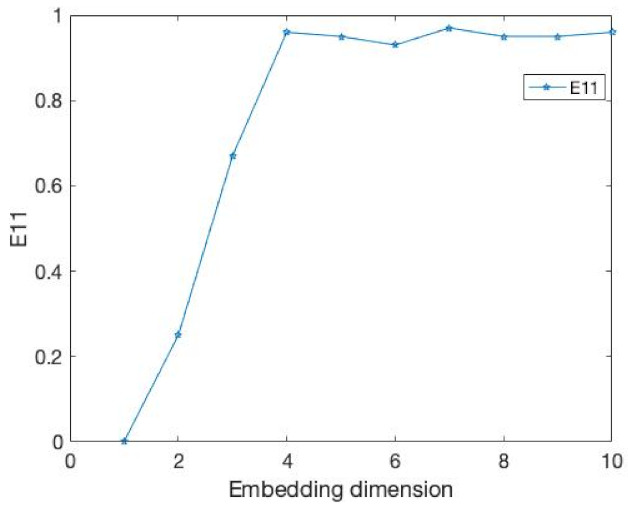
It is same as the Figure 3 but the value is E11.

The results of the comparison experiments are shown in Figure 3 and Figure 4 and Table 2.

In this example, the time delay is 1. Obviously, our E11 measure becomes stable after the embedding dimension reaches 4. At the same time, the DET value exceeds 0.9 and tends towards 1. This demonstrates that our method can well identify the embedding dimension of this time series. At the same time, the FNN method is where $A_{tol}=3,R_{tol}=9$ cannot accurately identify its embedding dimension when faced with a time series of 1000 data points.

Next, we are going to discuss the stability and applicability for time series from a high-dimensional attractor. Until the present, many methods only discussed the time series that is from low-dimensional systems; thus, the method that can determine the embedding dimension needs to be improved. As is demonstrate in [10], Cao tested the data which from Mackey-Glass delay-differential equation, as followed, has a high-dimensional attractor, but the result is unsatisfactory.

(17) $$\begin{array}{c} \frac{dx(t)}{dt}=-0.1x\left(t\right)+\frac{0.2x(t-\Delta )}{1+x{\left(t-\Delta \right)}^{10}}. \end{array}$$

**Figure 5:**
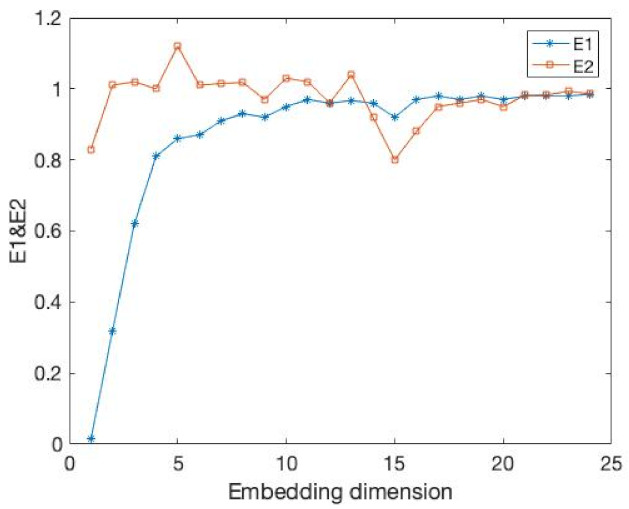
It is same as the Figure 2 but the data comes from Mackey-Glass delay-differential equation.

**Figure 6:**
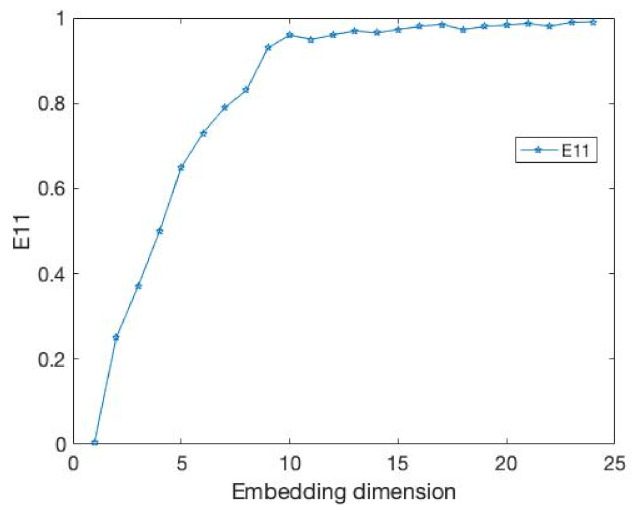
The value E11 for the data, which is same as the above data.

As demonstrated in Figure 5, there is a sudden dip at d=15; however, they could not explain the phenomenon and hope to investigating it in their future work. Thus, in order to solve this problem, we use our measure E11 and DET, which from the modified method tests the above Mackey-Glass series, and obtains the result in Figure 6 and Table 3.

Cao’s method has better advantages than the traditional FNN method. It can identify whether the time series is random data through the quantity E2. However, it is only useful for identification.

**Figure 7:**
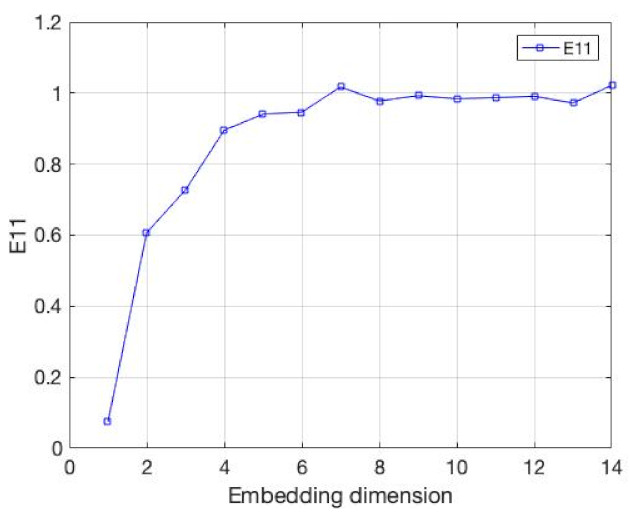
The value of E11, whose data are from the CSI 500 Index from 2016 to 2017.

**Figure 8:**
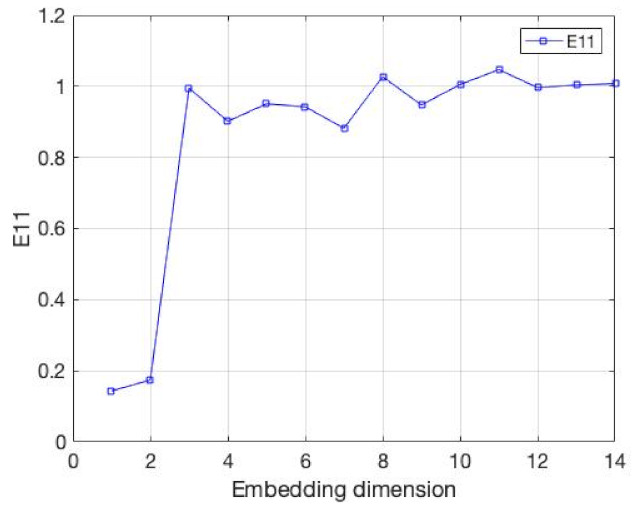
The value of E11, whose data are from the CSI 500 Index from 2018.

**Figure 9:**
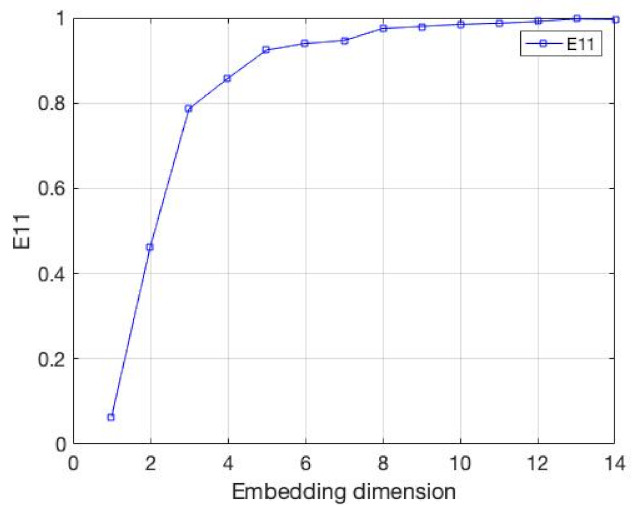
The value of E11, whose data are from the CSI 500 Index from 2019 to 2021.

Next, we will explore the role of the E11 indicator in the method. We selected the CSI 500 Index from 2016 to 2021 as the experimental data. Before the experiment, considering the impact of the market adjustment in 2018 on the embedding dimension of the time series, we divided the experimental data into three parts: before, during and after 2018, and analyzed the embedding dimension, respectively. The experimental results are shown in Figure 7, Figure 8 and Figure 9. The embedding dimension of the time series around 2018 reached the minimum around 5–6, while in 2018, due to the market adjustment, the minimum embedding dimension appeared around 3–4. This demonstrates that the market adjustment has an impact on the embedding dimension of the whole time series. There are 33–40% changes here, which indicates that the market adjustment has a significant impact on the embedding dimension of the whole time series; thus, the embedding dimension of the whole time series is not constant. Next, we will explore the advantages of the new method on the experimental data.

**Figure 10:**
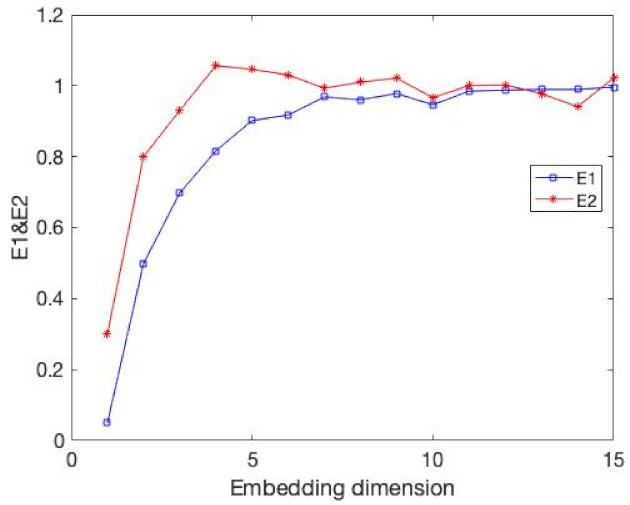
The value of E1, E2 whose data are from the CSI 500 Index from 2016 to 2021.

In our experiments, we found that when E1 tends to stabilize, E2 does not help us to determine when the embedded dimension stops increasing. Among them, we selected the daily closing chaotic time series of the CSI 500 Index from 2016 to 2021 as the experimental data. The experimental results are shown in Figure 10. The quantity of E1 becomes stable when the embedding dimension is 5 to 6; however, the quantity of E2 tends to be stable after the embedding dimension is 3.

**Figure 11:**
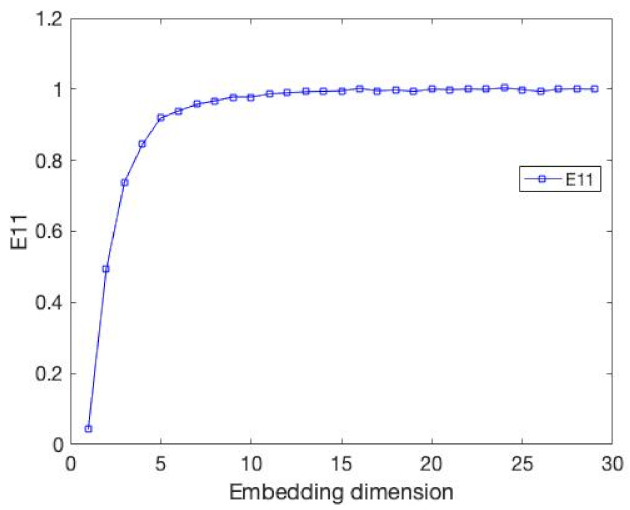
The value of E11, whose data are same with the above Figure 10.

We improved this part of the shortcomings by using the quantity DET of RQA. Through experiments on the above data, we obtained the experimental results, as shown in Figure 11 and Table 4. As shown in Figure 11, the value of quantity E11 becomes stable when the embedding dimension reaches 4 to 6. From Table 4, we can find that the value of quantity DET exceeds 0.9 after the embedding dimension reaches 5, and the certainty becomes very high. Therefore, we can effectively determine the optimal embedding dimension of the time series through quantity DET.

## 4. Conclusions

Aiming at the shortcomings of traditional methods for finding the embedding dimension of the time series, we propose a new and effective method based on them and perfect the financial time series. In addition, through the experimental analysis of the time series, we find that this method has better advantages in accuracy, stability, and calculation of the theoretical and financial time series with high-dimensional chaotic attractors. Financial systems are complex and full of systemic risk and nonlinear characteristics.

In the further study, we also hope that our new method will be useful in applications of nonlinear techniques to explore more financial time series as well as the artificial time series. Next, we will deal with the non-stationary data in a stable way, and our work analyzes the multi-scale and multi-domain financial time series to more accurately reveal the dynamic characteristics of the financial system.

## Figures and Tables

**Table 1:** The value DET for time series which is from the Lorenz attractor.

Dimension	DET
1	0.48
2	0.79
3	0.93
4	0.92
5	0.95
6	0.92
7	0.94
8	0.93
9	0.92

**Table 2:** The value DET for time series which is from the above map in Equation (14).

Dimension	DET
1	0.37
2	0.67
3	0.78
4	0.95
5	0.955
6	0.963
7	0.95
8	0.97
9	0.96
10	0.975

**Table 3:** The value DET for time series, which is from the Mackey-Glass delay-differential equation.

Dimension	DET	Dimension	DET
1	0.48	11	0.94
2	0.59	12	0.95
3	0.63	13	0.93
4	0.72	14	0.96
5	0.78	15	0.94
6	0.82	16	0.943
7	0.84	17	0.945
8	0.85	18	0.943
9	0.87	19	0.953
10	0.95	20	0.97

**Table 4:** The value DET for time series, which is same as Figure 10.

Dimension	DET
1	0.57
2	0.83
3	0.89
4	0.96
5	0.954
6	0.965
7	0.95
8	0.97
9	0.963

## References

[1] Takens F. (1981). Detecting strange attractors in turbulence. LNM.

[2] Sun B.J., Li M., Zhang F.F. (2019). The characteristics and self-time-delay synchronization of two-time-delay complex Lorenz system. J. Franklin Inst..

[3] Bhavsar R., Davey N., Helian N. (2018). Time Series Analysis using Embedding Dimension on Heart Rate Variability. Procedia Comput. Sci..

[4] Jazayeri M., Ostojic S. (2021). Interpreting neural computations by examining intrinsic and embedding dimensionality of neural activity. Curr. Opin. Neurobiol..

[5] Li D.Y., Cao M.S. (2021). A novel embedding method for characterization of low-dimensional nonlinear dynamical systems. Nonlinear Dyn..

[6] Sugihara G., May R.M. (1990). Applications of Fractals in Ecology. TREE.

[7] Machado J.A.T. (2014). Relativistic time effects in financial dynamics. Nonlinear Dyn..

[8] Machado J.A.T., Duarte F.B., Duarte G.M. (2011). Analysis of financial data series using fractional Fourier transform and multidimensional scaling. Nonlinear Dyn..

[9] Zhou Q., Zhu Z., Xian G. (2022). A novel regression method for harmonic analysis of time series. ISPRS J. Photogramm. Remote Sens..

[10] Karlaftis M.G., Vlahogianni E.I. (2009). Memory properties and fractional integration in transportation time-series. TR_C.

[11] Zolotova N.V., Ponyavin D.L. (2006). Phase asynchrony of the north-south sunspot activity. Astron. Astrophys..

[12] Liu W., Wang D.Z., Chen Z.H. (2017). Recurrence plot-based dynamic analysis on electrochemical noise of the evolutive corrosion process. Corros. Sci..

[13] King G.P., Stewart L. (1992). Phase space reconstruction for symmetric dynamical systems. Physica D.

[14] Atay F.M., Yurtsever E. (1997). Phase-space reconstruction in Hamiltonian systems through multiple time series. Chem. Phys. Lett..

[15] Wallot S., Monster D. (2018). Calculation of average mutual information (AMI) and false-nearest neighbors (FNN) for the estimation of embedding parameters of multidimensional time series in matlab. Front. Psychol..

[16] Kennel M.B., Brown R., Abarbanel H.D.I. (1992). Determine embedding dimension for phase-space reconstruction using a geometrical construction. Phys. Rev. A.

[17] Cao L.Y. (1997). Practical method for determining the minimum embedding dimension of a scalar time series. Physica D.

[18] Eckmann J.P., Kamphorst S.O., Ruelle D. (1987). Recurrence plots of dynamical systems. Europhys. Lett..

[19] Meng X.J., Qiu S., Wan S.H. (2021). A motor imagery EEG signal classification algorithm based on recurrence plot convolution neural network. Pattern Recognit. Lett..

[20] Kok T.L., Aldrich C. (2019). Analysis of Electrochemical Noise for Corrosion Type Identification by Use of Global Recurrence Plots and Texture Analysis. IFAC-Pap. OnLine.

[21] Webber C.L., Marwan N. (2015). Recurrence Quantification Analysis-Theory and Best Practices.

[22] Wallot S. (2019). Multidimensional cross-recurrence quantification analysis (MDCRQA)—A method for quantifying correlation between multivariate time-series. Multivar. Behav. Res..

[23] Xu M., Shang P.J., Lin A.J. (2017). Multiscale recurrence quantification analysis of order recurrence plot. Physica A.

[24] Marwan N., Wessel M., Kurths J. (2002). Recurrence Plot Based Measures of Complexity and its Application to Heart Rate Variability Data. Phys. Rev. E.

[25] Belaire J., Contreras D., Tordera L. (2002). Assessing nonlinear structures in real exchange rates using recurrence plot strategies. Physica D.

[26] Yao C.Z., Lin Q.W. (2017). Recurrecnce plots analysis of the CNY exchange markets based on phase space reconstruction. N. Am. J. Econ. Financ..

[27] Yin Y., Shang P.J. (2016). Multiscale recurrence plot and recurrence quantification analysis for financial time series. Nonlinear Dyn..

[28] Afonso L.C.S., Rosa G.H., Pereira C.R., Weber S.A.T., Hook C., Albuquerque V.H.C., Papa J.P. (2019). A recurrence plot-based approach for Parkinson’s disease identification. Future Gener. Comput. Syst..

[29] Zbilut J.P., Webber C.L. (1992). Embeddings and delays as derived from quantification of recurrence plots. Phys. Lett. A.

[30] Tielen G.J., Luek T., Kuzma M. (1997). The role of Manhattan distance in antiferromagnetic ordering. Physica A.

[31] Gueye S., Michelon P. (2015). A linear formulation withvarianles for quadratic assignment problems with Manhattan distance matrices. EURO J. Comput..

[32] Blackburn S.R., Homberger C., Winkler P. (2019). The minimum Manhattan distance and minimum jump of permutations. J. Comb. Theory Ser. A.

[33] Neyman S.N., Sitohang B., Sutiusna S. (2013). Reversible Fragile Watermarking based on Difference Expansion Using Manhattan Distances for 2D Vector Map. Procedia Technol..

